# 1778. Same Story, Different Outbreak: Lessons from a Survey Study Investigating JYNNEOS Mpox Public Vaccination Clinic Establishment in New Hampshire

**DOI:** 10.1093/ofid/ofad500.1607

**Published:** 2023-11-27

**Authors:** HeeEun Kang, Rattanaporn Mahatanan, Devin Lee, Stephanie L Locke, Elizabeth A Talbot, Benjamin P Chan

**Affiliations:** Dartmouth Hitchcock Medical Center, Lebanon, New Hampshire; Dartmouth-Hitchcock Medical Center, Lebanon, New Hampshire; Dartmouth Health, Wailuku, Hawaii; NH Division of Public Health Services, Concord, New Hampshire; Geisel School of Medicine at Dartmouth, Lebanon, NH; NH Department of Health and Human Services, Concord, New Hampshire

## Abstract

**Background:**

The 2022-2023 mpox outbreak necessitated rapid distribution of JYNNEOS vaccines from the US Strategic National Stockpile to protect at-risk persons. New Hampshire’s centralized public health structure requires partnership with healthcare facilities to administer vaccines during public health responses. We reached out to 67 organizations; only seven were able to establish a community JYNNEOS vaccine clinic.

**Methods:**

To identify barriers and resources needed for emergency public vaccination, surveys were sent to seven participating and 20 non-participating organizations.

**Results:**

Seven (100%) participating organizations (“vaccine-partners”) and seven (35%) non-participating organizations responded. Two out of seven non-participating respondents were non-clinical and lacked facilities and staff to conduct vaccination clinics. Of the remaining five non-participating respondents, all identified lack of staffing, provider time, and clinical resources as barriers to establishing community vaccination clinics. Vaccine-partners and non-participating organizations reported needing or utilizing the following staff positions to conduct public vaccination clinics: administrators (0.15-1.5 Full Time Equivalents [FTE]), physicians or advanced practice providers (0.2-1 FTE), registered nurses (0.17-1.5 FTE), medical/licensed nursing assistants (0.2-1 FTE), and communications outreach staff (0.5 FTE). In addition to staffing, additional funding needs estimated by all survey respondents averaged $2,057 per month (range $0-$7,500).

**Figure 1.**
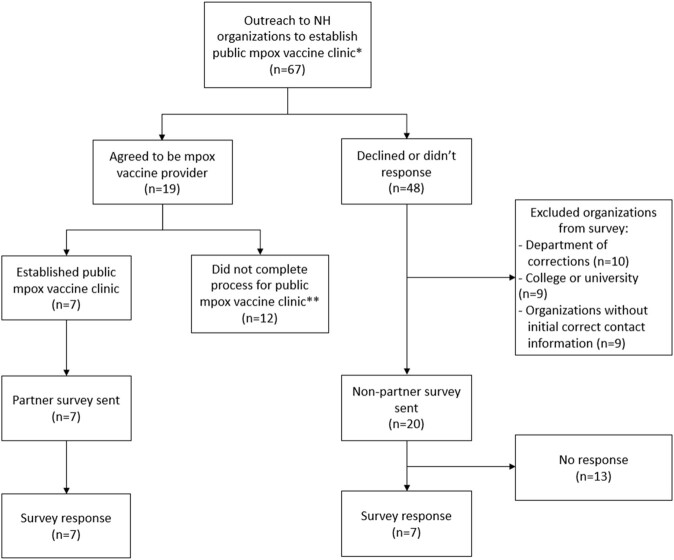
Consort flow diagram. *Public mpox vaccination clinics were intended to be clinics open to the general public (i.e., open points of distribution, or PODS) serving both the organizations existing patient population, new patients, and referrals from other providers or public health for both post-exposure prophylaxis (PEP) and pre-exposure prophylaxis (PrEP) vaccination. Public mpox vaccination clinics were advertised on the NH DHHS website and in the media as a resource for all NH residents and providers. ** These clinics went through initial "onboarding" process to be able to receive and administer mpox vaccine, but they either didn't complete the process to be a public clinic, only served as a PEP provider as needed, or only chose to administer vaccine to their patient population which was allowed in select circumstances (e.g., with FQHCs) to increase equitable access.

**Figure 2.**
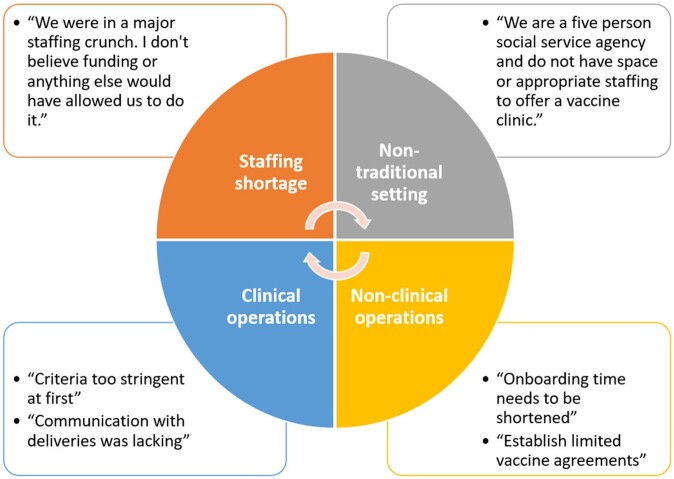
Barriers to establishing public mpox vaccine clinic

**Conclusion:**

A minority of NH healthcare facilities elected to establish public JYNNEOS vaccination clinics. Insufficient staffing was the primary barrier. Additional resources and funding were minimal. The repeated need for public health vaccination clinics requires sustained advocacy, support, and partnership to ensure an efficient and effective emergency response for the next outbreak.

**Disclosures:**

**Benjamin P. Chan, MD, MPH**, General Electric (GE): Stocks/Bonds

